# Impact of age at onset on symptom profiles, treatment characteristics and health-related quality of life in Parkinson’s disease

**DOI:** 10.1038/s41598-021-04356-8

**Published:** 2022-01-11

**Authors:** Lars Lau Raket, Daniel Oudin Åström, Jenny M. Norlin, Klas Kellerborg, Pablo Martinez-Martin, Per Odin

**Affiliations:** 1 H. Lundbeck A/S, Valby, Denmark; 2 Clinical Memory Research Unit, Department of Clinical Sciences, Lund University, Lund, Sweden; 3 The Swedish Institute for Health Economics, Lund, Sweden; 4 Center of Networked Biomedical Research in Neurodegenerative Diseases (CIBERNED), Carlos III Institute of Health, Madrid, Spain; 5 Division of Neurology, Department of Clinical Sciences, Lund University, Lund, Sweden; 6 Forskningsenhet Neurologi, Skåne University Hospital, Wigerthuset, Remissgatan 4, pl 1, 222 42 Lund, Sweden

**Keywords:** Basal ganglia, Neurological disorders

## Abstract

Parkinson’s disease (PD) is typically considered an age-related disease, but the age at disease onset can vary by decades between patients. Aging and aging-associated diseases can affect the movement system independently of PD, and advanced age has previously been proposed to be associated with a more severe PD phenotype with accelerated progression. In this work, we investigated how interactions between PD progression and aging affect a wide range of outcomes related to PD motor and nonmotor symptoms as well as Health Related Quality of Life (HRQoL) and treatment characteristics. This population-based cohort study is based on 1436 PD patients from southern Sweden followed longitudinally for up to approximately 7.5 years from enrollment (3470 visits covering 2285 patient years, average follow-up time 1.7 years). Higher age at onset was generally associated with faster progression of motor symptoms, with a notable exception of dyskinesia and other levodopa-associated motor fluctuations that had less severe trajectories for patients with higher age at onset. Mixed results were observed for emergence of non-motor symptoms, while higher age at onset was generally associated with worse HRQoL trajectories. Accounting for these identified age-associated differences in disease progression could positively impact patient management and drug development efforts.

## Introduction

Parkinson’s disease (PD) is typically considered an age-related disease^1^. While age is often mentioned as the major risk factor, the age at disease onset can vary by decades^2^. Both in its prodrome and after disease onset, clinical and pathological milestones have been identified that seem to occur with some regularity^3^. A range of symptoms that are associated with PD, including motor and cognitive symptoms, may also arise due to aging and co-pathologies, and age at symptom onset or age at diagnosis have previously been reported to influence the course of disease^4–8^. Further to this, it has been proposed that aging-associated co-pathologies are not only additive, but may interact with the PD-pathology to produce a more severe phenotype than what one would expect from the individual contributions of disease progression and aging^9^.

While there is strong evidence that PD manifestation is influenced by two major timescales, namely age and disease duration (e.g. years since onset of motor symptoms), the mechanisms and resulting effects have only been explored to a limited extent. Simple hypotheses such as monotone decline in clinical scores along with both age, disease duration and their interaction may not be well grounded. This is for example illustrated by the fact that known pathogenic genetic variants have been found to affect both age at onset, rate of disease progression, and likelihood of occurrence of specific symptoms^10^, which points to the biological complexity of disease manifestation. Furthermore, while higher age is generally associated with a higher load of non-PD specific pathology^11^, a higher age at onset of PD could potentially also indicate that the patient has been in better general health prior to disease^12^ or has experienced less detrimental exposures. In these cases, one could imagine that older age at onset would lead to a slower progression of PD, all other things being equal.

In this paper, we explore the different timescales of PD by analyzing the evolution of symptom scores, clinical scale components, health-related quality of life (HRQoL) instruments, and medication use along aging and disease duration.

## Materials and methods

### The Swedish National Parkinson Register, PARKreg

The Swedish National registry for Parkinson’s Disease (PARKreg), which was established in 2011 with the aim to contribute to Swedish Parkinson care through continuous follow-up of clinical measures and HRQoL instruments in clinical practice and through research. The register includes demographic variables, diagnosis, treatments, and physician reported clinical measures of disease severity, and patient reported outcomes. Information is included per clinical practice when the patients visit the doctor/nurse at the neurologist’s office, which is at least once a year. Due to the longitudinal nature of the study, patients were not necessarily assessed by the same neurologist at all visits. The registry currently covers at least one registration in approximately 6800 patients out of approximately 22 000 patients with Parkinson´s disease in Sweden. This study is based on data on patients from the southernmost region of Sweden, Scania, with idiopathic Parkinson’s disease in PARKreg that was retrieved in April 2020.

The Scania Cohort of PARKreg has an estimated coverage of approximately 50% of patients diagnosed with PD in the region. Included patients were required to have at least one valid assessment of one of the study outcomes apart from treatment variables.

### Treatment data

Data on prescriptions and pick-ups of treatment was linked to the PARKreg Cohort by a unique personal identification number using data from the Swedish Prescribed Drug Register, maintained by the National Board of Health and Welfare. Medications in the register had associated defined daily dose data that describe the assumed average maintenance dose per day for the medication when used for its main indication in adults. Daily doses were calculated as the number of defined daily doses picked up divided by pick up frequency (i.e. a patient picking up a prescription with a total of 7 defined daily doses every week would be assigned a daily dose of 1).

Levodopa prescriptions were considered any prescription with ATC code in the category *N04BA Dopa and dopa derivatives*. Average daily levodopa dose was computed for each half year between first and last registered pick up. Daily dose at any time was computed by linear interpolation of the half-yearly average doses. Before first registered pick up, the dose was set to 0. After the last half-year mark before the last pick up, daily doses were set to the last average half-yearly dose until all subsequent picked-up doses would run out, and after that the daily dose was set to 0.

Average Levodopa Equivalent Daily Dose (LEDD) of all prescribed medications was computed for each patient. This computation was done following the above description for average daily levodopa dose where for prescriptions outside of N04BA, conversion factors from the Parkinson's Measurement Levodopa Equivalent Dose Calculator^13^ were used to convert to the LEDD scale.

Current and former (ever or last 6 months) treatments with drugs in any direct sub-categories of ATC code *N04B DOPAMINERGIC AGENTS* were included as outcomes. If a sub-category contained at least two individual drugs that each had more than 10.000 registered pickups, each of these drugs were also included as separate outcomes.

### Study variables and outcomes

The study examined different time variables and their relation to PD progression. The five main time variables considered were age at PD onset, age at PD diagnosis, age at baseline, years since PD onset, and years since PD diagnosis. PD onset was defined as self-reported onset of motor symptoms while time variables related to age and diagnosis were defined based on central registers.

This study included symptom-based outcomes, HRQoL outcomes, and treatment outcomes. The symptom-based outcomes were investigated using the following scales; Clinical Impression of Severity Index for Parkinson's Disease (CISI‐PD)^14^ (six-item scale (0–6) with a total score ranging from 0–36, with higher scores indicating higher levels of disability), Modified Hoehn & Yahr staging (H&Y)^5,15^ (seven categories ranging from 0.0–5.0 with higher scores indicating higher levels of disability) and Non-Motor Symptom Questionnaire (NMSQ)^16^ (30-item scale with a total score ranging from 0–30 with higher scores indicating higher non-motor symptom load). In addition, dystonia and daily dystonia time were evaluated along with freezing of gait and off fluctuations. The HRQoL outcomes were investigated using the Eight-item Parkinson’s Disease Questionnaire (PDQ-8)^17^ and the EQ-5D-3 level version (EQ-5D-3L), including the EQ-5D-3L visual analogue scale (VAS)^18^. Treatment outcomes (such as active prescriptions and doses) were extracted using data on filled prescriptions. The treatment outcomes included average daily levodopa dose, averaged LEDD and number of different dopaminergic agents (ATC prefix N04B) the patient had been exposed to (last 6 months/ever) at a given visit. Current or former use of individual drugs or drug types within the group of dopaminergic agents were also considered as outcomes at baseline following the convention described in the previous section.

A detailed description of the outcomes scales is available in the Supplementary material.

### Statistical analysis

Spearman correlations were used to evaluate the relation between symptom scores and scale components at baseline and the five considered time variables, age at PD onset, age at PD diagnosis, age at baseline, years since PD onset, and years since PD diagnosis. Years on dopaminergic treatment were highly correlated with years since PD diagnosis, correlation coefficient 0.83, and thus not further included in the analyses.

Longitudinal trajectories of outcomes were analyzed using linear mixed-effects models. The models all assume that unobserved data is missing at random. Outcome trajectories were modeled by fixed-effect natural splines over both age and time since onset. For each model, the degrees of freedom for each spline basis (between 1 and 5) were selected using the Schwarz Bayesian Information Criterion (BIC). All models had a random intercept, slope and second-order polynomial term in time since baseline describing the within-patient time variation. A free covariance structure between the random coefficients was assumed. For each outcome, four distinct models of the temporal evolution of outcomes were considered and the one with the lowest BIC was selected as the final model. The first model only included effects of age, the second model only included effects of time since onset, the third model included additive effects of age and time since onset, and the fourth model included interactions between age and time since onset. The interpretation of the first and second models is that the outcomes are not affected by disease progression and age, respectively. The interpretation of the third model (additive) is that aging and disease progression affect outcomes independently, while the interpretation of the fourth model (interaction) is that the outcome is affected synergistically by an interactions between aging and disease progression that give rise to different outcomes than what one would expect from the individual estimated contributions. All statistical analyses were done using R 4.0.2^19^. Mixed-effects models were fit using the lme4 package^20^.

### Ethics

The study was conducted in compliance with the Helsinki Declaration. Participants in the Parkinson registry are included after giving their informed consent. All participants were able to withdraw from the registry at any point in time. This study was approved by the Swedish Ethical Review Authority on 4. Feb 2020 (Decision number 2019-05791).

## Results

This study included 1436 PD patients with at least one valid assessment of one of the non-treatment outcomes. Data on prescriptions and pick-ups included 205,463 records classified as anti-parkinson drugs (ATC code N04). The average number of visits per patient was 2.4 (standard deviation (sd) 1.5) and the average and median follow-up times were 1.7 and 0.3 years with an interquartile range of [0, 3.4]. The demographics and patient characteristics are given in Table 1. The median age at baseline was 72 years and the patients had a median duration of 4.2 years since diagnosis and 6.2 years since onset of motor symptoms. The disease status of the patients was relatively mild, with approximately three quarters of patients having unilateral or mild bilateral involvement at baseline according to H&Y stage I or II. Most patients (90.6%) were treated with levodopa at the time of their baseline visit.

**Table 1:** Patient characteristics and follow up.

	N	
Male—N (%)	1436	900 (62.7%)
Birth year—median [IQR]		1944 [1939, 1949]
Age at baseline—median [IQR]		72 [66, 77]
Age at onset—median [IQR]	1256	65 [57, 71]
Age at diagnosis—median [IQR]	1317	67 [59, 73]
Years since onset—median [IQR]	1256	6.2 [3.7, 10.7]
Years since diagnosis—median [IQR]	1317	4.2 [1.6, 8.6]
Relatives with PD—N (%)	1251	238 (19.0%)
Levodopa treatment at baseline—N (%)	1436	1301 (90.6%)
Levodopa-naïve at baseline—N (%)	1436	58 (4.0%)
**Hoehn and Yahr score at baseline—N (%)**	1320	
No signs of disease		10(0.8%)
Unilateral involvement only		3022.9%)
Unilateral and axial involvement		1370.4%)
Bilateral involvement without impairment of balance		2821.4%)
Mild bilateral disease with recovery on pull test		193 (14.6%)
Mild to moderate bilateral disease; some postural instability; physically independent		251 (19.0%)
Severe disability; still able to walk or stand unassisted		77 (5.8%)
Wheelchair bound or bedridden unless aided		22 (1.7%)
Number of visits—median [IQR]	1436	2 [1, 3]
Patients with only baseline visit—N (%)		516 (36%)
Follow up time—mean [SD]		1.7 [2.2]

### Baseline correlations

**Figure 1:**
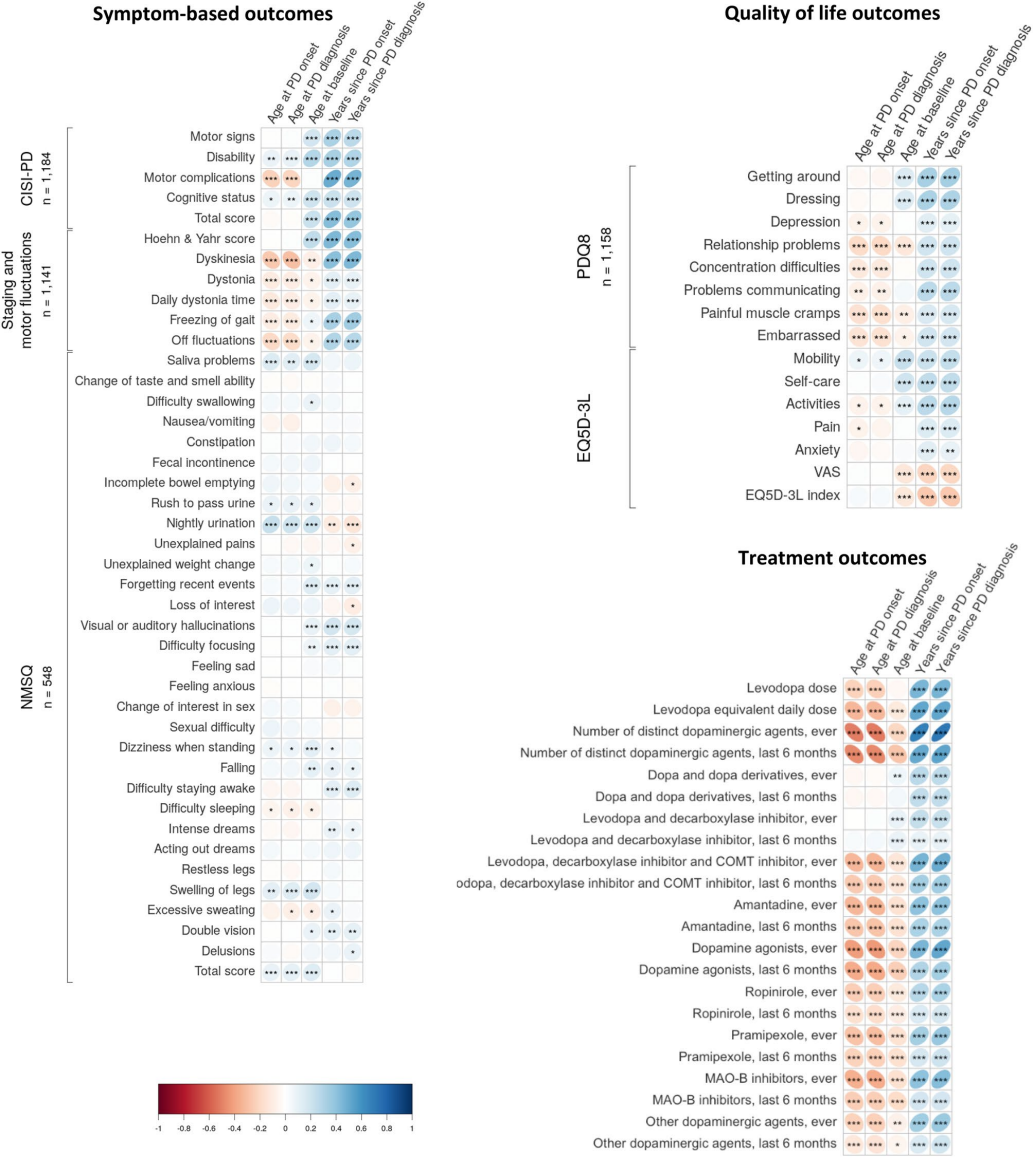
Baseline Spearman correlations between time variables (age at Parkinson’s Disease onset, age at Parkinson’s Disease diagnosis, age at baseline, years since Parkinson’s Disease onset, years since Parkinson’s Disease diagnosis), and respectively, symptom-based outcomes, Health related Quality of Life outcomes, and treatment outcomes.

The correlations between the individual outcomes and time variables are shown in Fig. 1. Due to the high correlation between time at onset of motor symptoms and time at diagnosis within patient, almost identical correlations for variables anchored in these two milestones were observed across outcomes. Therefore, we will focus on time variables related to the onset milestone in the following.

For the symptom-based outcomes, the largest correlations were observed for CISI-PD items and outcomes relating to staging and motor fluctuations, while the non-motor symptom outcomes captured by NMSQ generally only had modest correlations to the time variables. The numerically largest correlations to time since onset were seen for CISI-PD motor complications (0.49), H&Y score (0.45), CISI-PD total score (0.42), and dyskinesia (0.42); all *p* < 0.0001. The largest correlations to age were seen for CISI-PD disability (0.28), H&Y score (0.25), CISI-PD total score (0.22), and CISI-PD cognitive status (0.21); all *p* < 0.0001. The largest correlations to age at onset were dyskinesia (-0.27), NMSQ nightly urination (0.23), CISI-PD motor complications (-0.22), off fluctuations (-0.19); all *p* < 0.0001.

For the outcomes related to HRQoL, the largest correlations to time since onset were the PDQ8 items *getting around* (0.32), *dressing* (0.31), and *activities* (0.27), followed by the EQ5D-3L index (-0.27); all *p* < 0.0001. The largest correlations to age were seen for EQ5D-3L mobility (0.23) and EQ5D-3L self-care (0.19); both *p* < 0.0001. The largest correlations to age at onset were PDQ8 relationship problems (-0.18) and PDQ8 painful muscle cramps (-0.15); all *p* < 0.0001.

For treatment outcomes, the largest correlations to time since onset were number of distinct dopaminergic agents tried, ever/last 6 months (0.67/0.50) and LEDD (0.51); all *p* < 0.0001, suggesting that longer disease duration was associated with a wider variety of treatments tried and higher LEDD. The largest correlations to age were seen for number of distinct dopaminergic agents tried, last 6 months (-0.28) and use of dopamine agonists, last 6 months/ever (-0.24/-0.21); all *p* < 0.0001, suggesting that older patients were less exposed to different dopaminergic agents in the 6 months leading up to baseline and generally less exposed to dopamine agonists. The largest correlations to age at onset were number of distinct dopaminergic agents tried, ever/last 6 months (-0.50/-0.48), followed by use of dopamine agonists, last 6 months/ever (-0.41/-0.37); all *p* < 0.0001, suggesting that patients with higher age at onset, like patients with higher age at baseline, were less exposed to different dopaminergic agents and less use of dopamine agonists.

### Longitudinal trajectories

**Figure 2:**
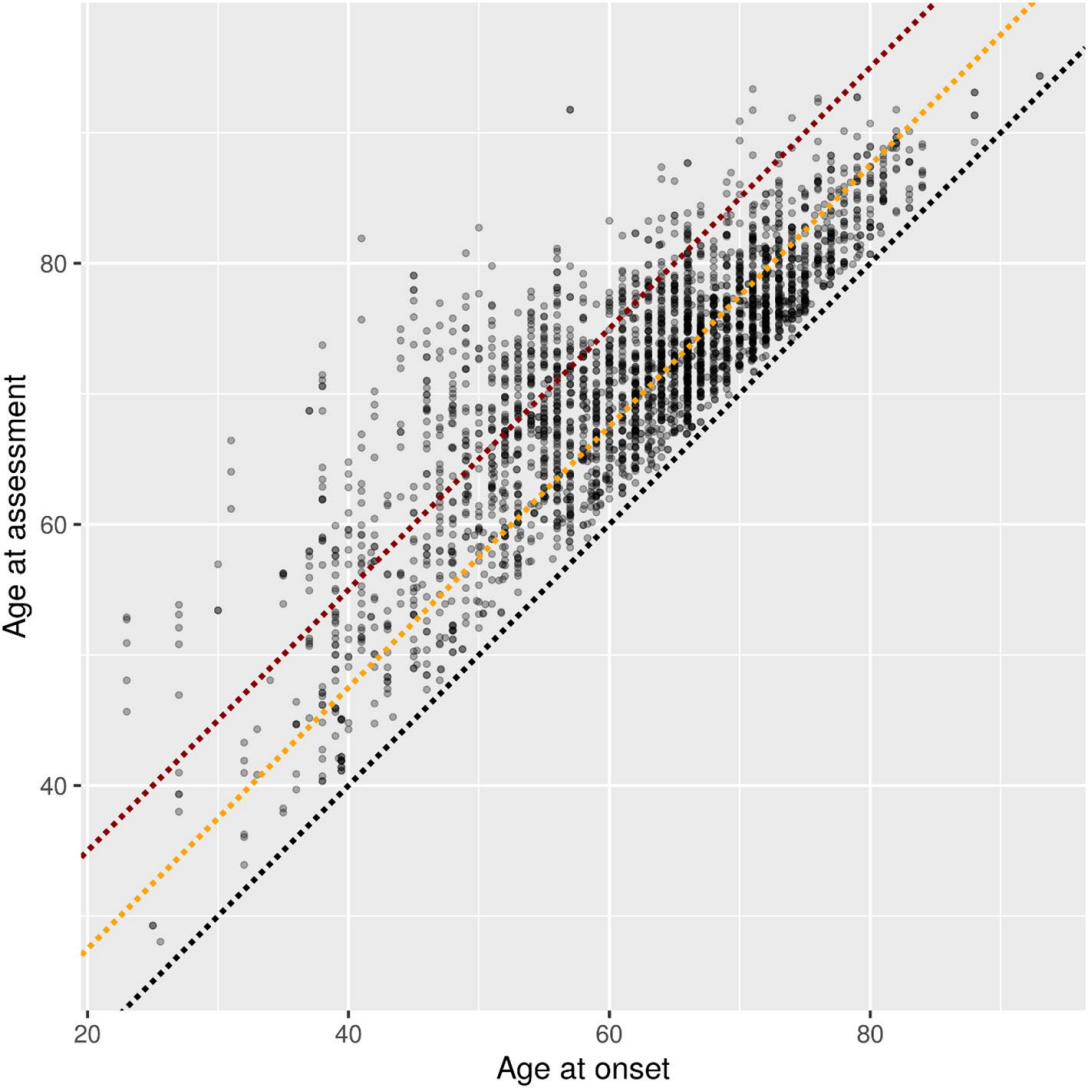
Patient ages at study visits against patient age at onset. The black line represents age at onset, the orange line represents 7.5 years after onset, and the dark red line represents 15 years after onset.

Patient ages at every study visit are plotted against the patient age at onset in Fig. 2. This figure shows that there is good data coverage at least 15 years following onset for onset ages between 45 and 75.

**Figure 3:**
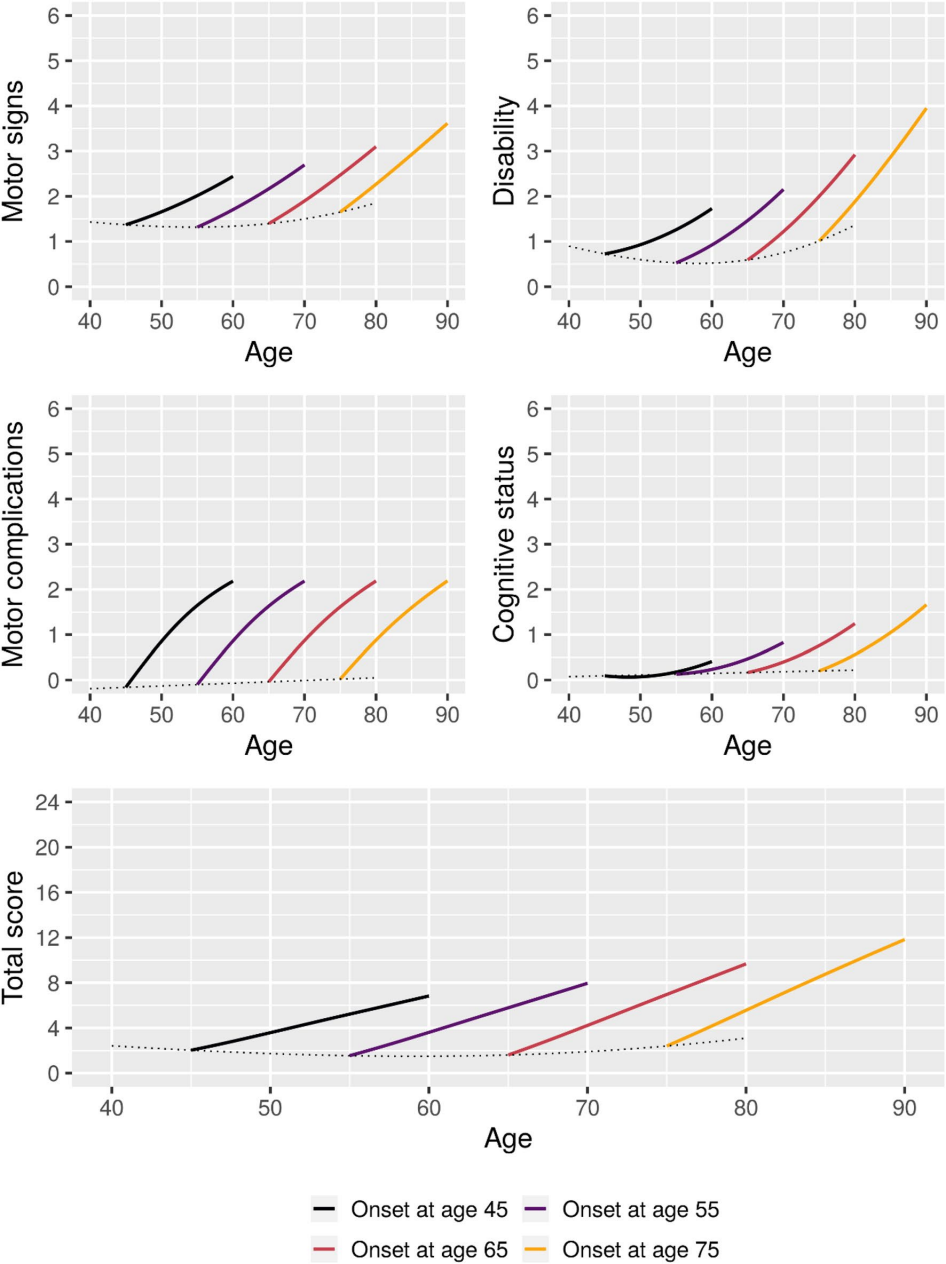
Estimated 15-year mean trajectories of CISI-PD outcomes from Parkinson’s disease onset at respectively 45, 55, 65, and 75 years of age. Trajectories estimated based on data from 2298 visits. Dotted line represents the estimated mean scores for patients with onset at the given age.

**Figure 4:**
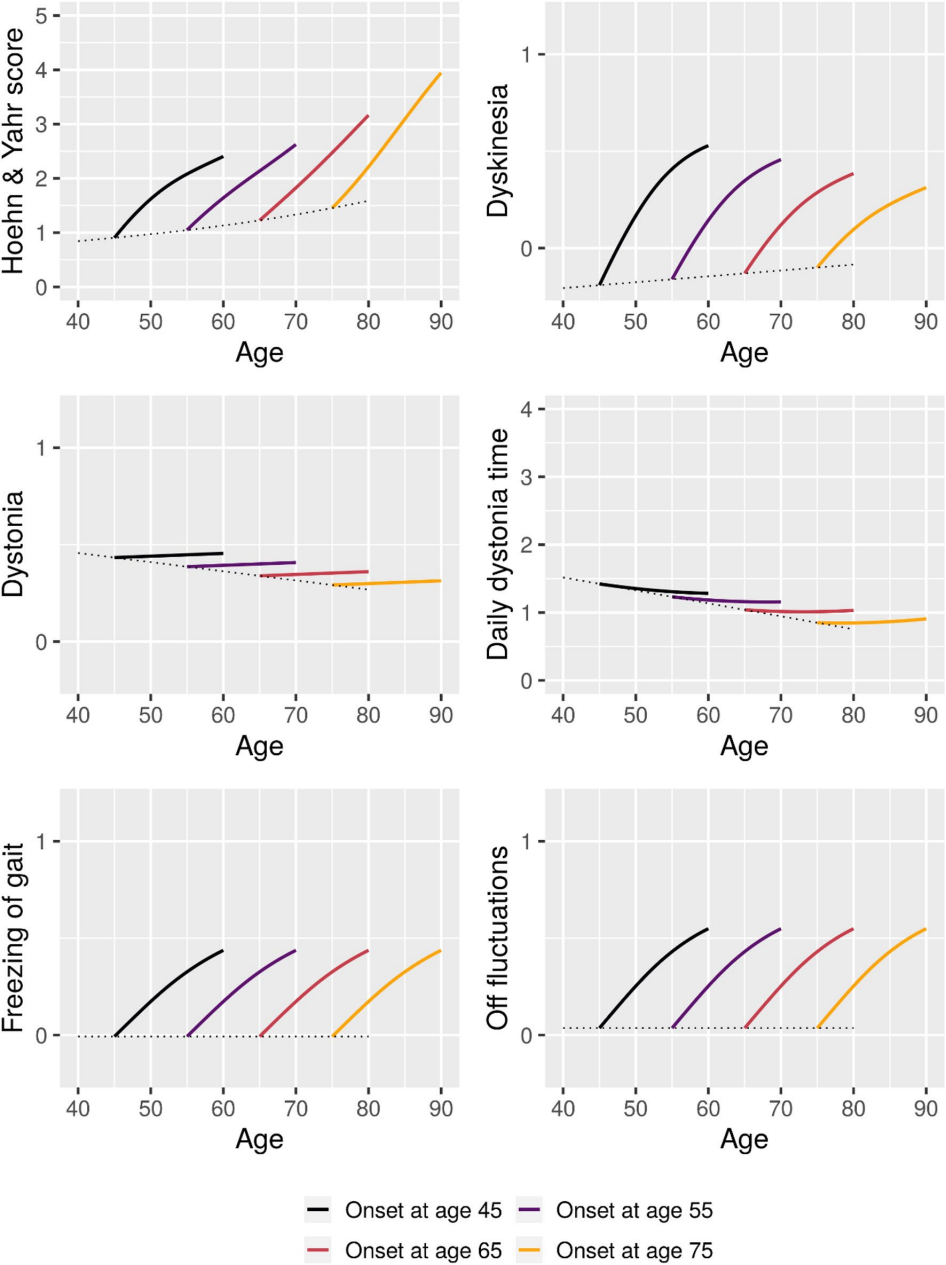
Estimated 15-year mean trajectories of staging and motor fluctuation outcomes from Parkinson’s disease onset at respectively 45, 55, 65, and 75 years of age. Trajectories estimated based on data from 2307 visits (Hoehn & Yahr) and 2510 visits (motor fluctuation outcomes). Dotted line represents the estimated mean scores for patients with onset at the given age.

**Figure 5:**
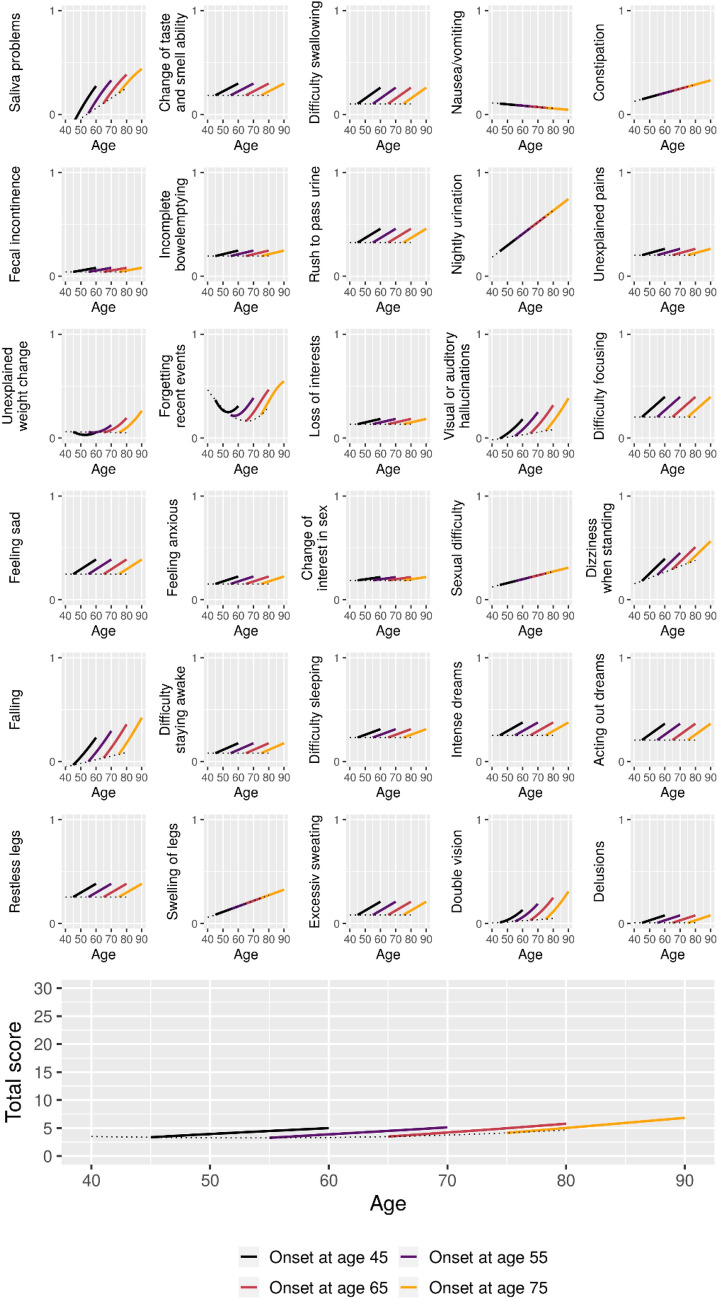
Estimated 15-year mean trajectories of Non-Motor Symptom Questionnaire outcomes from Parkinson’s disease onset at respectively 45, 55, 65, and 75 years of age. Trajectories estimated based on data from 2758 visits. Dotted line represents the estimated mean scores for patients with onset at the given age.

**Figure 6:**
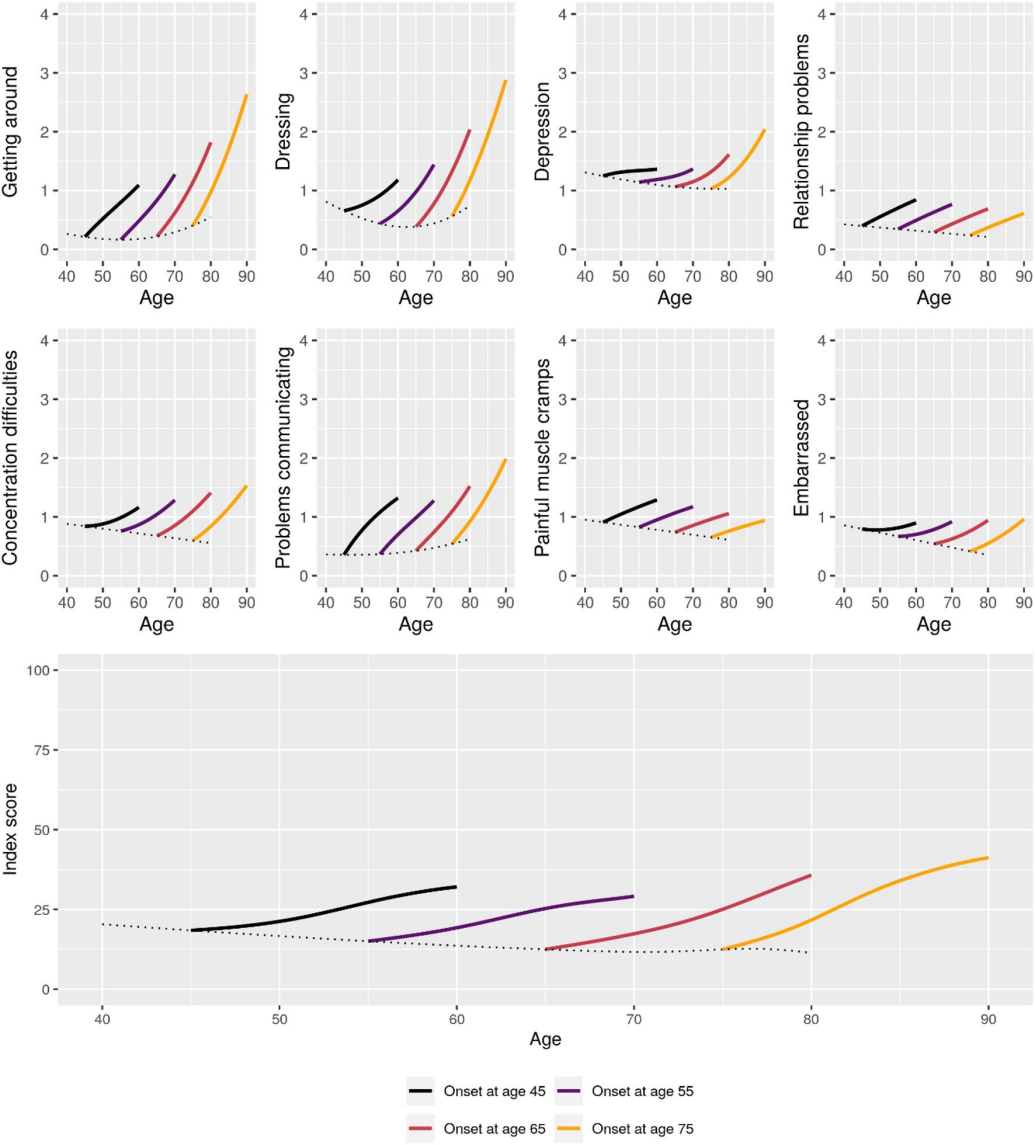
Estimated 15-year mean trajectories of 8-item Parkinson’s Disease Questionnaire outcomes from Parkinson’s disease onset at respectively 45, 55, 65, and 75 years of age. Trajectories estimated based on data from 2421 visits. Dotted line represents the estimated mean scores for patients with onset at the given age.

Estimated 15-year longitudinal trajectories of symptom-based outcomes for patients with onset at ages 45, 55, 65, and 75 are shown in Figs. 3, 4, 5, 6.

All selected models for CISI-PD outcomes included interactions between time since onset and age, albeit the contribution of the interaction effect for Motor complications was very small. In general, higher age at onset was associated with faster progression.

For staging and motor fluctuation outcomes, interactions were seen for H&Y score where later onset was associated with faster progression, for Dyskinesia where later onset was associated with less dyskinesia and for Daily dystonia time, where the contribution of the interaction effect was small. For Dystonia, main effects of both time since onset and age were found where later onset was associated with less dystonia, but symptom presence only showed very limited change with the progression of disease. Both Freezing of gait and Off fluctuations were found to become more frequent with the progression of disease, but no effect of age was found.

For the NMSQ total score, an interaction between time since onset and age was seen, such that later onset was associated with slightly more nonmotor symptoms and more symptoms appearing over time. For the individual NMSQ outcomes, interactions were only seen for six outcomes (Saliva problems, Unexplained weight change, Forgetting recent events, Visual or auditory hallucinations, Falling, Double vision) where higher age at onset was in general associated with worse outcomes. Dizziness when standing was the only outcome showing additive main effects of age and time since onset without an interaction. Five outcomes (Nausea/vomiting, Constipation, Nightly urination, Sexual difficulty, Swelling of legs) did not show any association with progression of disease. The remaining outcomes (Change of taste and smell ability, Difficulty swallowing, Fecal incontinence, Incomplete bowel emptying, Rush to pass urine, Unexplained pains, Loss of interest, Difficulty focusing, Feeling sad, Feeling anxious, Change of interest in sex, Falling, Difficulty staying awake, Difficulty sleeping, Intense dreams, Acting out dreams, Restless legs, Excessive sweating, Delusions) all showed higher incidence of the symptoms with disease progression without an effect of age.

**Figure 7:**
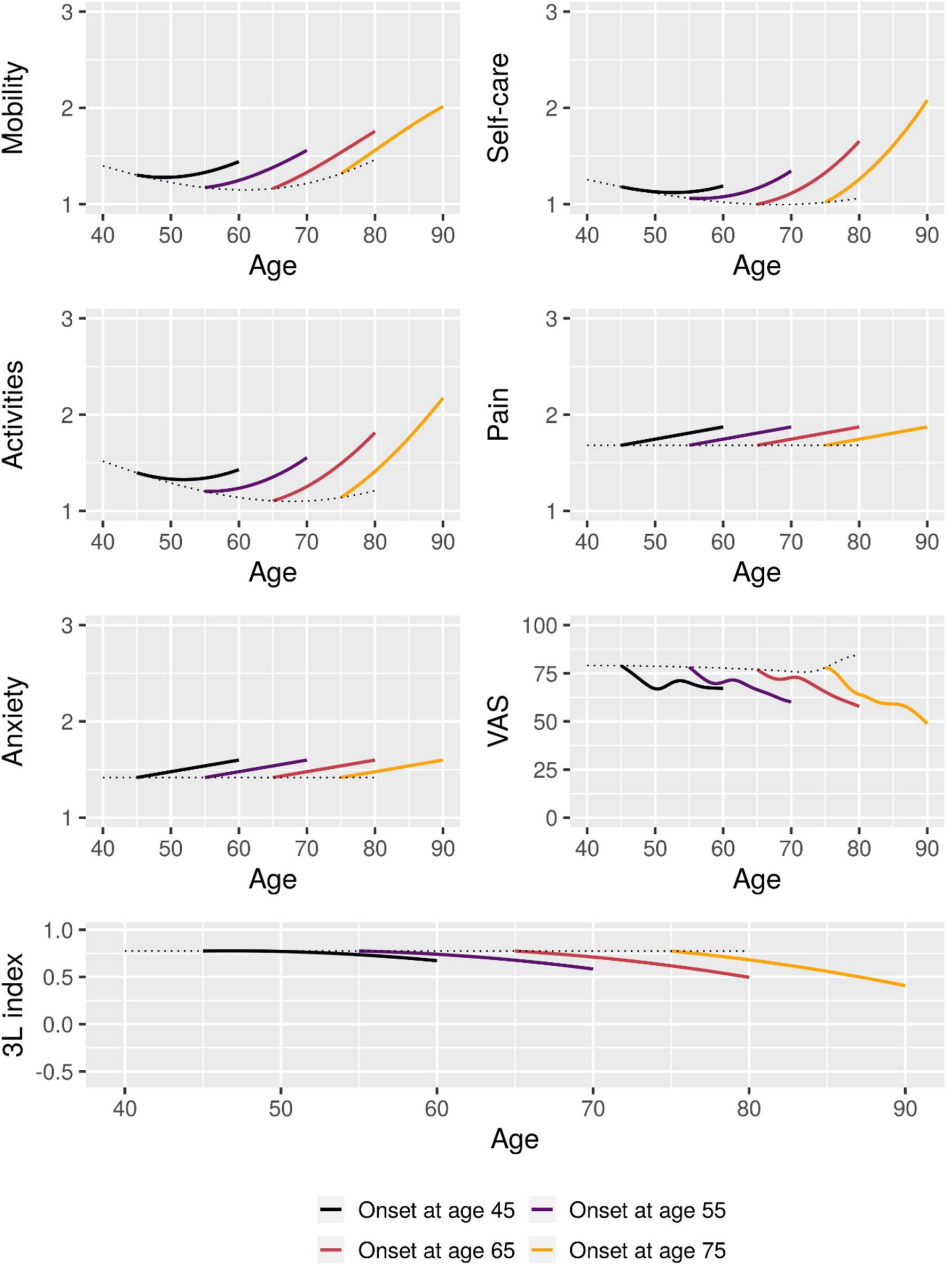
Estimated 15-year mean trajectories of EQ5D-3L outcomes from Parkinson’s disease onset at respectively 45, 55, 65, and 75 years of age. Trajectories estimated based on data from 1800 visits. Dotted line represents the estimated mean scores for patients with onset at the given age.

Estimated 15-year longitudinal trajectories of HRQoL outcomes for patients with onset at ages 45, 55, 65, and 75 are shown in Figs. 6, 7.

All selected models for PDQ-8 outcomes included interactions between time since onset and age. For most of the outcomes, a negative trend in age was seen around age at onset, suggesting that earlier onset was associated with lower HRQoL or a greater initial impact on HRQoL than later onset. However, later onset was associated with faster worsening of HRQoL as measured by most of the outcomes.

For EQ-5D-3L outcomes, Mobility, Self-care, Activities, VAS and the 3L index all included interactions. In general, higher age at onset was associated with faster deterioration in HRQoL. The VAS did display somewhat complex trajectories. Neither pain nor anxiety showed any effects of age but did show an increasing burden with disease progression.

**Figure 8:**
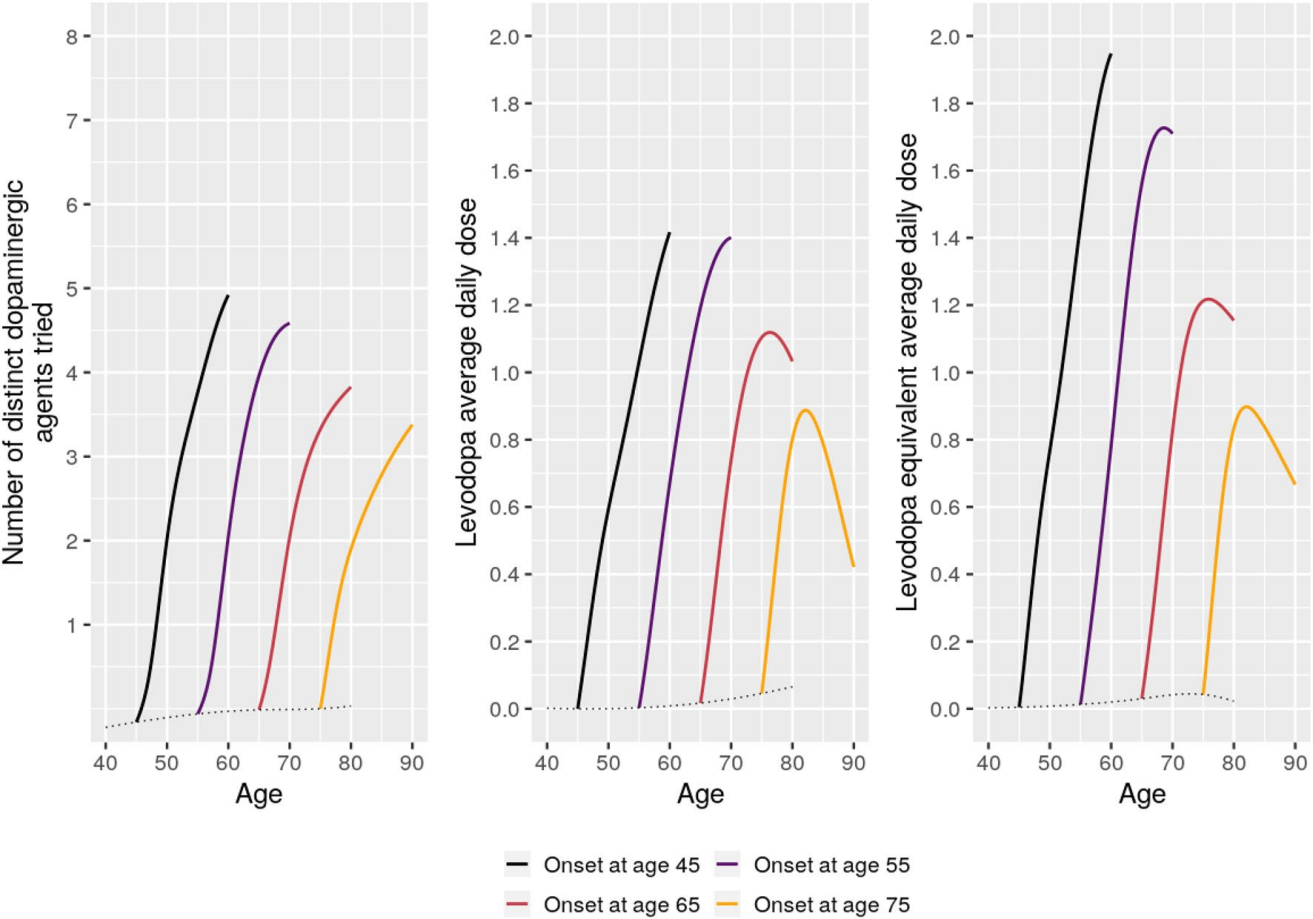
Estimated 15-year mean trajectories of treatment outcomes from Parkinson’s disease onset at respectively 45, 55, 65, and 75 years of age. Trajectories estimated based on data from 205,463 prescriptions and pick-ups mapped to 3407 visits. Dotted line represents the estimated mean scores for patients with onset at the given age.

Estimated 15-year longitudinal trajectories of treatment outcomes for patients with onset at ages 45, 55, 65, and 75 are shown in Fig. 8. Both number of distinct dopaminergic agents tried, and average daily levodopa dose showed interactions between time since onset and age. Higher age at onset was associated with less exposure to different dopaminergic agents as disease progressed and with less increase (and eventual decrease) in daily levodopa dose.

## Discussion

The manifestation of Parkinson’s disease is a complex and heterogeneous. In the present population-based cohort study, we investigated how symptom profiles, treatment characteristics and HRQoL were affected by the interplay between aging and disease progression. We found that progression in many outcomes seemed to be affected by interactions between these two timescales, and when interactions were seen, it was typically such that higher age at onset was associated with a more severe course of disease. The two notable exceptions to this were measures of dyskinesia and dystonia, where higher age at onset was associated with less compared to patients with earlier onset. These exceptions are in line with previous findings indicating that lower age at onset is predictive of time to wearing off and time to dyskinesia^21^.

Understanding the factors that drive heterogeneity of disease presentation is useful both on the individual level and for clinical research. In particular, when defining subpopulations of patients, it is important for the grouping factors to be simple, implementable and interpretable^22^, which is certainly the case for age at onset. On the individual level, prognostic information can be useful for guiding treatment choices and for planning for the future, which is identified as a key need for patients and care partners^23,24^. For drug development, knowledge of factors that affect longitudinal outcomes can be used to increase the statistical power of studies; either by adjusting efficacy analyses for these factors, or by enriching studies by restricting inclusion to patients that are likely to have a more homogeneous progression. Furthermore, knowing that higher age at onset generally associated with worse outcomes may suggest that other age-related pathologies interact with the Lewy-body pathology to produce a more severe phenotype. This in turn could indicate that drug candidates targeting PD pathology such as alpha synuclein antibodies could have a relatively lower efficacy in patients with a higher age at onset.

Analyses of associations between HRQoL and motor/non-motor symptoms suggests significant contributions to PD burden^25^. Regarding temporal changes in HRQoL in this large and well-defined PD cohort, we found increasingly impaired HRQoL over time, similar to other findings in the literature. Reuther and colleagues^22^ and Visser and colleagues^23^ both reported decreased HRQoL as measured by EQ-5D over a 12- and 24-month follow-up period. In a Scandinavian setting Karlsen and colleagues^26^ reported similar findings. However, these studies were limited in cohort size as well as follow-up time.

Strengths of this study includes its size, the population-based cohort, the longitudinal study design, and the ability to link patients to the Swedish Prescribed Drug Register to accurately capture their use of medication. The study also has some weaknesses regarding the data as the coverage of patients diagnosed in the region is approximately 50% with patients with advanced disease being underrepresented. Furthermore, the study did not include the Unified Parkinson's disease rating scale^27^, a common endpoint in interventional clinical trials, and thus we could not investigate the potential impact of aging and disease progression interaction on the typical primary outcome measure of clinical trials. Another weakness related to the outcomes was the use of NMSQ that only asses presence of symptoms and not changes in symptom severity or frequency, which are captured with the Non-Motor Symptom Scale^28^. Finally, the analyses did not assess the potential effects of other comorbidities.

## Supplementary Information

Supplementary Information.

## Data Availability

The data that support the findings of this study are available from the corresponding author upon reasonable request. The fitted model objects describing the longitudinal trajectories and estimated variance parameters are available for download at https://github.com/larslau/PD_progression/.
