# Supplementary material for: Impact of age at onset on symptom profiles, treatment characteristics and health-related quality of life in Parkinson’s disease
Source: Sci Rep. 2022 Jan 11;12:526. doi: 10.1038/s41598-021-04356-8 (PMC8752787; doi:10.1038/s41598-021-04356-8)
Supplement: Supplementary file 1 — Supplementary Information. [file 41598_2021_4356_MOESM1_ESM.docx]

*Supplementary Material 1.*

*Symptom-based outcomes*

Clinical Impression of Severity Index for Parkinson's Disease (CISI‐PD)^11^ included the following variables

- Motor signs
- Disability
- Motor complications
- Cognitive symptoms
- Total score

Staging and motor fluctuations included the following variables

- Modified Hoehn & Yahr staging (H&Y)^5,12^
- Dystonia
- Daily dystonia time
- Freezing of gait
- Off fluctuations

Non-Motor Symptom Questionnaire (NMSQ)^13^ included the following variables

- Saliva problems
- Change of taste and smell ability
- Difficulty swallowing
- Nausea/vomiting
- Constipation
- Fecal incontinence
- Incomplete bowel emptying
- Rush to pass urine
- Nightly urination
- Unexplained pains
- Unexplained weight change
- Forgetting recent events
- Loss of interest
- Visual or auditory hallucinations
- Difficulty focusing
- Feeling sad
- Feeling anxious
- Change of interest in sex
- Sexual difficulty
- Dizziness when standing
- Falling
- Difficulty staying awake
- Difficulty sleeping
- Intense dreams
- Acting out dreams
- Restless legs
- Swelling of legs
- Excessive sweating
- Double vision
- Delusions
- Total score

*Quality of Life outcomes*

Eight-item Parkinson’s Disease Questionnaire (PDQ8)^14^ included the following variables

- Getting around
- Dressing
- Depression
- Embarrassed
- Relationship problems
- Concentration difficulties
- Unable to communicate
- Painful muscle cramps

EuroQoL-5 domains-3 levels (EQ5D-3L)^15^ included the following variables

- Mobility
- Self-care
- Activities
- Pain
- Anxiety
- VAS
- EQ5D-3L index (Dolan tariff)

*Treatment outcomes*

Treatment outcomes (such as active prescriptions and doses) were extracted using data on filled prescriptions. The treatment outcomes at a given visit included

- Number of different dopaminergic agents (ATC prefix N04B) the patient had been exposed to up to this visit
- Levodopa average daily dose
- Levodopa equivalent average daily dose
